# Reorganization of the 3D chromatin architecture of rice genomes during heat stress

**DOI:** 10.1186/s12915-021-00996-4

**Published:** 2021-03-19

**Authors:** Zhe Liang, Qian Zhang, Changmian Ji, Guihua Hu, Pingxian Zhang, Yifan Wang, Liwen Yang, Xiaofeng Gu

**Affiliations:** 1 Biotechnology Research Institute, Chinese Academy of Agricultural Sciences, Beijing, 100081 China; 2 Centre for Organismal Studies, Heidelberg University, 69120 Heidelberg, Germany; 3 Institute of Tropical Bioscience and Biotechnology, Chinese Academy of Tropical Agricultural Sciences, Haikou, 571101 China

**Keywords:** Hi-C, Rice, Heat stress, TAD, Plant, Chromatin organization, ATAC-seq, A/B compartment

## Abstract

**Background:**

The three-dimensional spatial organization of the genome plays important roles in chromatin accessibility and gene expression in multiple biological processes and has been reported to be altered in response to environmental stress. However, the functional changes in spatial genome organization during environmental changes in crop plants are poorly understood.

**Results:**

Here we perform Hi-C, ATAC-seq, and RNA-seq in two agronomically important rice cultivars, Nipponbare (Nip; *Japonica*) and 93-11 (*Indica*), to report a comprehensive profile of nuclear dynamics during heat stress (HS). We show that heat stress affects different levels of chromosome organization, including A/B compartment transition, increase in the size of topologically associated domains, and loss of short-range interactions. The chromatin architectural changes were associated with chromatin accessibility and gene expression changes. Comparative analysis revealed that 93-11 exhibited more dynamic gene expression and chromatin accessibility changes, including HS-related genes, consistent with observed higher HS tolerance in this cultivar.

**Conclusions:**

Our data uncovered higher-order chromatin architecture as a new layer in understanding transcriptional regulation in response to heat stress in rice.

**Supplementary Information:**

The online version contains supplementary material available at 10.1186/s12915-021-00996-4.

## Background

Spatial organization of the three-dimensional (3D) genome plays crucial roles in gene transcription regulation and controlling multiple biological processes in eukaryotic organisms [1–3]. Chromosome conformation capture technologies, such as Hi-C, have been developed as powerful tools for 3D genome analysis and have revealed several global and local levels of chromatin organization, including (1) megabase-scale A and B compartments that are related to active and inactive gene expression, respectively [4]; (2) sub-megabase-scale domain, called topologically associating domains (TADs), which are 3D structural basic units that are separated from each other and are important for transcription regulation and replication [5]; and (3) chromatin loops, such as enhancer-promoter interactions that are directly involved in transcriptional regulation [6].

In plants, 3D chromatin organization maps have been reported in model *Arabidopsis* and several crops, including rice, maize, barley, and tomato [7–15], and have revealed that 3D chromatin architectures are highly correlated with the functionality of the genome. The impact of environmental changes on 3D chromatin organization has been reported in few eukaryotic species [16–18]. However, how the 3D chromatin changes in response to stress remains largely elusive in crops. In this study, we used Hi-C to examine the dynamics of the 3D genome during heat stress in two rice (*Oryza sativa*) cultivars Nip and 93-11. Our result suggests that heat stress affects A/B compartment transition, TAD size, and long-range interactions. By integrating Hi-C, RNA-seq, and ATAC-seq, we found that chromatin organization changes are associated with chromatin accessibility and gene expression changes. Interestingly, we found that 93-11 exhibited more dynamic gene expression and chromatin accessibility changes than Nip, coinciding with higher HS tolerance in 93-11 [19]. Taken together, our data uncovered 3D chromatin architecture as a new layer in understanding transcriptional regulation in response to heat stress in crops.

## Results and discussion

### Chromatin 3D structure dynamics in response to heat stress in rice

**Fig. 1:**
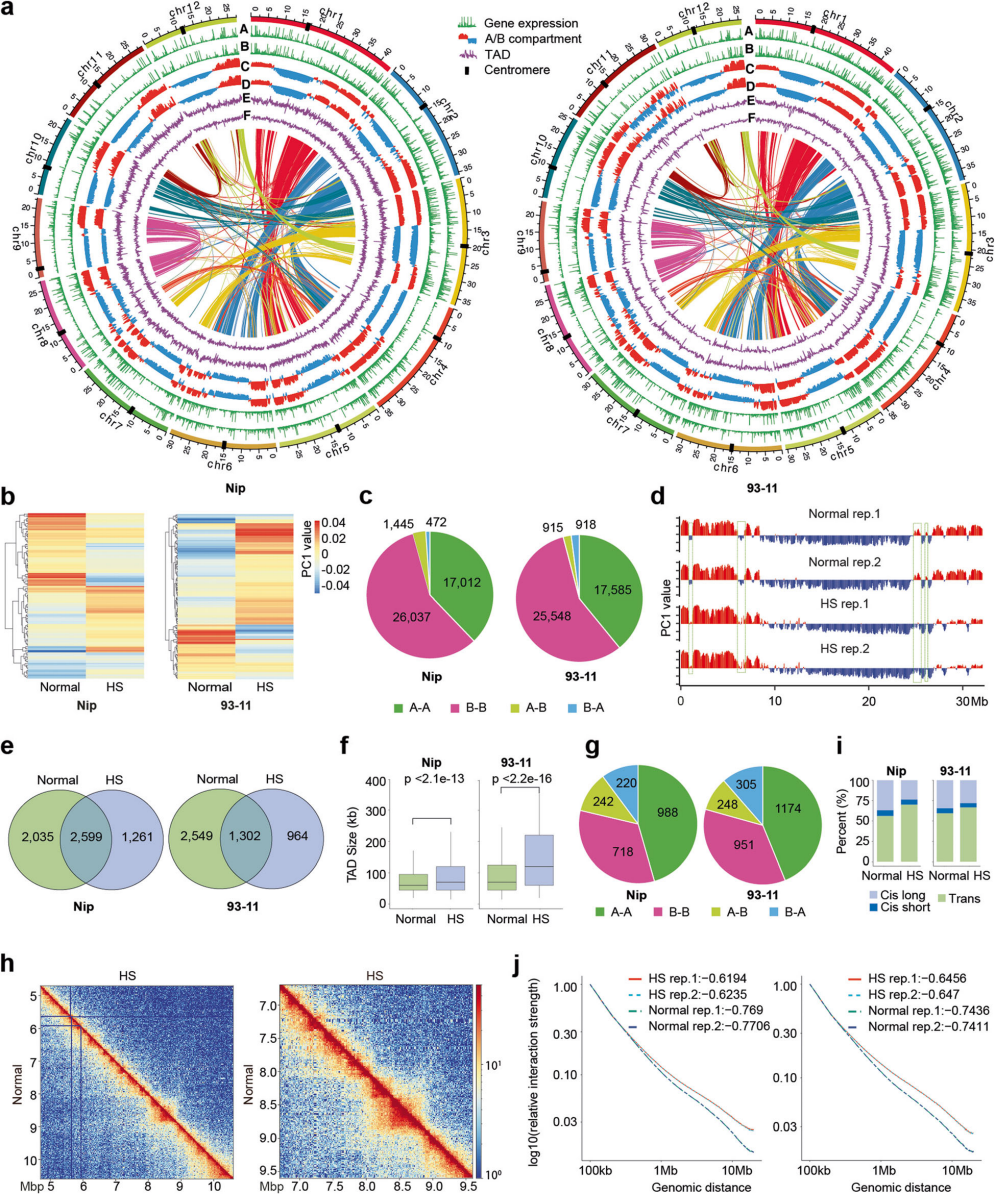
Chromatin 3D structure dynamics in response to heat stress in rice Nip and 93-11. **a** Circos plots of the chromatin 3D structure of Nip (left panel) and 93-11 (right panel) genomes. Seven rings from the outside to inside show genomic positions (outmost, black dots marked centromeres), gene expression FPKM (fragments per kilobase per million mapped reads) value for normal (**a**) and HS (**b**) samples, PC1 signal of A compartment (red) and B compartment (blue) for normal (**c**) and HS (**d**) samples, and TAD-separation score for normal (**e**) and HS (**f**) samples. A map connecting homologous regions of the genome is shown inside the figure. The colored lines link collinearity blocks that represent syntenic regions. **b** Heatmap of the PC1 scores of the HS-induced compartment switching regions in Nip and 93-11, respectively. **c** Global compartment numbers divided by stable (A-A and B-B) and switching (A-B and B-A) compartments upon HS in Nip and 93-11, respectively. **d** PC1 scores for chromosome 6 of 93-11, green boxes highlight regions transitioning from A to B and B to A upon HS. **e** Venn diagram showing the number of shared and sample-specific TADs in Nip and 93-11, respectively. The shared TADs were defined when 80% of the TAD were overlapped. **f** Boxplot comparing TAD size between normal and HS samples in Nip and 93-11, respectively. **g** In the regions that lost TAD boundaries upon HS, pie plot showing local compartment numbers divided by stable (A-A and B-B) and switching (A-B and B-A) compartments in Nip and 93-11, respectively. **h** Example of comparing of Hi-C interaction matrix from a region of chromosome 11 (5-kb resolution) in normal and HS samples in Nip. **i** Barplot comparing the percentage of different types of interactions between normal and HS samples in Nip and 93-11, respectively. Based on distance, interactions were classified to cis long (> 20 kb) and cis short (< 20 kb) and trans interactions. **j** Distance plot of interaction strength changes upon HS in Nip and 93-11, respectively

We first performed Hi-C experiments with two biological replicates from a 4-cutter restriction enzyme Dpn II digested aerial parts of 3-week-old Nip and 93-11 seedlings and obtained more than 130 million valid contacts in each. The Pearson correlation of chromatin interactions showed that replicates were largely correlated (Additional file 1: Fig. S1a,1b and Additional file 2: Table S1), suggesting high reproducibility of the Hi-C experiment. Long-range compartmentalization (A/B compartments) was examined using principal component analysis (PCA) of normalized Hi-C matrices. We identified A/B compartments in Nip and 93-11 and found that 82% of their genomes share synteny A/B compartments and their distribution patterns were highly conserved between Nip and 93-11 (Fig. 1a). The A compartment was more euchromatic, and the B compartment contained centromeres and pericentromeric regions, which was in line with the recent findings [7]. Of note, the A compartment displayed higher integrity of synteny pairs than that of the B compartment in both Nip and 93-11 (Fig. 1a). We further performed RNA-seq and compared the global gene expression activity between A and B compartments. As expected, a higher level of gene expression was observed in the A compartment (Fig. 1a), consistent with the association of the A compartment with open chromatin and the B compartment with closed chromatin. Next, we analyzed and compared the local level of chromatin organization represented by TADs and found that the number and distribution of TADs were quite similar between Nip and 93-11 (Fig. 1a and Additional file 3: Table S2). Taken together, these comparisons suggest a conserved chromatin interaction pattern between the two rice genomes.

To investigate the 3D structure dynamics in response to heat stress in rice, we performed Hi-C experiments from 3-week-old Nip and 93-11 aerial parts of seedlings under HS condition (Additional file 1: Fig. S1c). Comparative analysis revealed that HS changed the compartment and local chromatin organization in both Nip and 93-11 (Fig. 1a–j). We identified 1917 and 1833 transited A/B compartments following HS in Nip and 93-11, respectively (Fig. 1c), and these compartment reorganizations occurred in all chromosomes. Notably, most of these transitions were observed in smaller compartments or in part of the large compartment. Take the chromosome 6 of 93-11 as an example (Fig. 1d), analysis of the eigenvalues indicated that the first and second B compartments were surrounded by A compartments under the normal condition, and HS converted the two B compartment to A compartments and formed a larger A compartment (Fig. 1d).

Next, we analyzed the effects of HS on a more local level of chromatin organization. Using HiCExplorer [20], we identified 2943 and 2452 TADs in Nip and 93-11, respectively, in HS; these numbers are less than those (3112 and 2652) in the normal condition (Fig. 1e, Additional file 1: Fig. S1d and Additional file 3: Table S2). Venn diagram analysis revealed that only 44% (Nip) and 27% (93-11) of the total TADs are maintained under HS (Fig. 1e), suggesting that TADs are dramatically reorganized upon HS. Notably, the size of TADs was significantly increased following HS, particularly for the TADs within B compartments (Fig. 1f and Additional file 1: Fig. S2a). Most of the missed TAD boundaries induced by HS were located in the unchanged local A or B compartment (Fig. 1g), suggesting TAD dynamic may be independent from local A/B compartmentalization. To visualize and better understand these effects, we generated a reference contact matrix for regions in which TADs were affected upon HS (Fig. 1h). Although some borders were conserved, many TAD boundaries disappeared and the neighboring TADs were merged into larger ones upon HS (Fig. 1h). We then investigated the effects of HS on more local chromatin organization and found that HS largely affect chromatin interactions. The cis interactions, particularly the short-distance interaction, were significantly decreased during HS, while the trans interactions (between different chromosomes) were relatively increased (Fig. 1i, j and Additional file 1: Fig. S2b,c). We also observed that more loss occurred in A-A and B-B interactions than in A-B interactions (Additional file 1: Fig. S2d). Taken together, our data demonstrate a recompartmentalization of higher-order chromatin domains and local chromatin organization changed following HS in rice, which is in agreement with that HS inducing widespread rearrangement of 3D chromatin organization in *Arabidopsis* and *Drosophila* [17, 18].

### Chromatin 3D structure and gene expression are correlated upon HS in rice

**Fig. 2:**
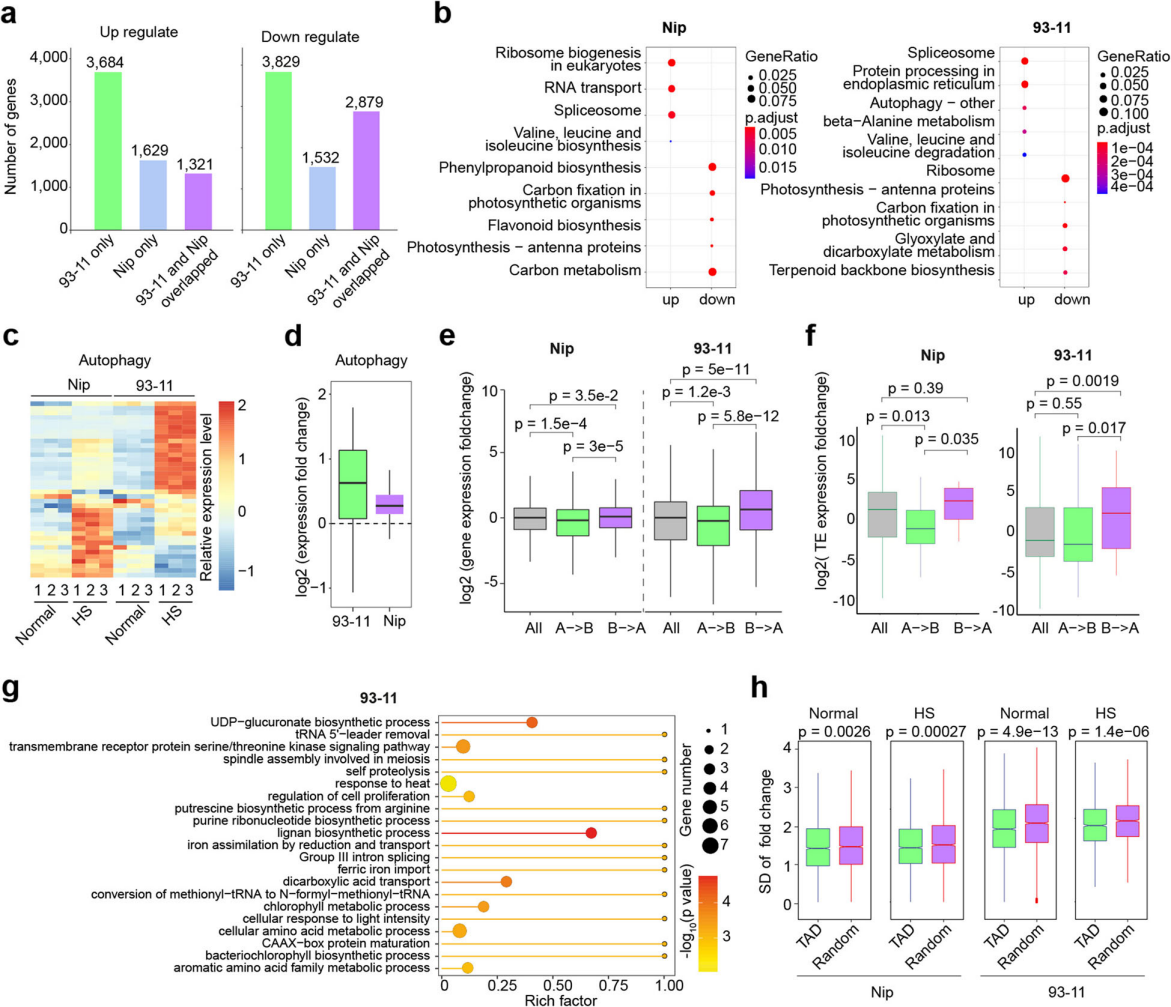
Chromatin 3D structure and gene expression are correlated upon HS in rice. **a** Comparison of the number of up- and downregulated genes that were divided into cultivar specific and overlapped between Nip and 93-11. **b** KEGG pathway enrichment analysis of DEGs upon HS in Nip and 93-11, respectively. **c**, **d** Heatmap (**c**) and box plot (**d**) showing change of expression of autophagy-related genes upon HS in Nip and 93-11, respectively. **e** Box plot comparing protein coding gene expression fold changes between genes in switch regions (A-B and B-A) and all genes (as a control) upon HS in Nip and 93-11, respectively. *p* values were calculated by performing a two-sided Wilcoxon test. **f** Box plot comparing TE expression fold changes between TE in switch regions (A-B and B-A) upon HS in Nip and 93-11, respectively. *p* values were calculated by performing a two-sided Wilcoxon test. **g** Scatter plots of significant biological processes as determined by GO enrichment analysis of DEGs in A-B or B-A transition regions upon heat stress in 93-11. **h** Box plot comparing standard deviation (SD) of gene expression fold change between genes in TAD interiors and randomly selected. *p* values were calculated by performing a two-sided Wilcoxon test

Compared with Nip, 93-11 exhibited greater HS tolerance [19] and more dynamic chromatin organization changes, including a higher TAD size changes in 93-11 than that in Nip (Fig. 1e, f). To investigate genes whose expression may be regulated in accompany with these changes, we performed RNA-seq with three replicates on RNA extracted from normal and HS-treated Nip and 93-11 aerial parts of seedlings, respectively (Additional file 1: Fig. S3a,b and Additional file 2: Table S3). We identified 3788 and 6262 upregulated, and 4971 and 7966 downregulated genes (fold change > 2, adjust *p* < 0.01) upon HS in Nip and 93-11, respectively (Additional file 2: Table S4 and S5). Similar to chromatin organization changes, 93-11 showed a higher number of differentially expressed genes (DEGs) than Nip (Fig. 2a). To provide functional insights into the DEGs, we performed KEGG pathway analysis and found that many KEGG terms were shared between Nip and 93-11, indicating HS affects several common pathways in rice, e.g., the downregulation of photosynthesis and synthesis of secondary metabolites. Interestingly, genes in the protein processing in ER and autophagy pathways, which facilitate the refolding and degradation of damaged proteins and related to HS [21], were upregulated in 93-11, but not in Nip (Fig. 2b–d), suggesting that a higher cellular capacity to maintain the proteostasis contributes to the heat adaptation of 93-11.

Comparison of DEGs with our list of genes located within the A/B compartment dynamic regions revealed 355 and 594 DEGs with altered A/B compartments in response to HS in Nip and 93-11, respectively. Although the overall overlapping was not significant (Additional file 1: Fig. S3c), genes in the B-A compartment transition regions were significantly upregulated, and genes in the A-B compartment transition regions were significantly downregulated (Fig. 2e). Similarly, we also found that expression of Transposable Elements (TEs) was significantly upregulated in the B-A compartment switch regions (Fig. 2f). These observations implicate that A/B compartmentalization changes strongly affect gene expression upon HS in both Nip and 93-11, and also in line with that A compartment is associated with open chromatin. Moreover, we found that upregulation (B-A) or downregulation (A-B) for DEGs was more significant in 93-11 than that in Nip (Fig. 2e, f), consistent with that 93-11 exhibited more dynamic chromatin changes. Gene Ontology (GO) analysis focusing on those genes in regions with A/B compartment transition showed high enrichment for genes involved in heat stress in 93-11 (Fig. 2g). However, the heat stress-related GO terms were not found for the same type of DEGs in Nip (Additional file 1: Fig. S3d), suggesting that chromatin organization changes associated with HS-related gene expression changes in 93-11, but not in Nip. In addition, we compared the standard deviation (SD) of gene expression fold change and found that SD were significantly lower for the genes within TAD interiors than those of the randomly selected ones (Fig. 2h), suggesting that genes in the same TAD tend to be more co-regulated.

### Chromatin 3D structure and chromatin accessibility are correlated in rice

**Fig. 3:**
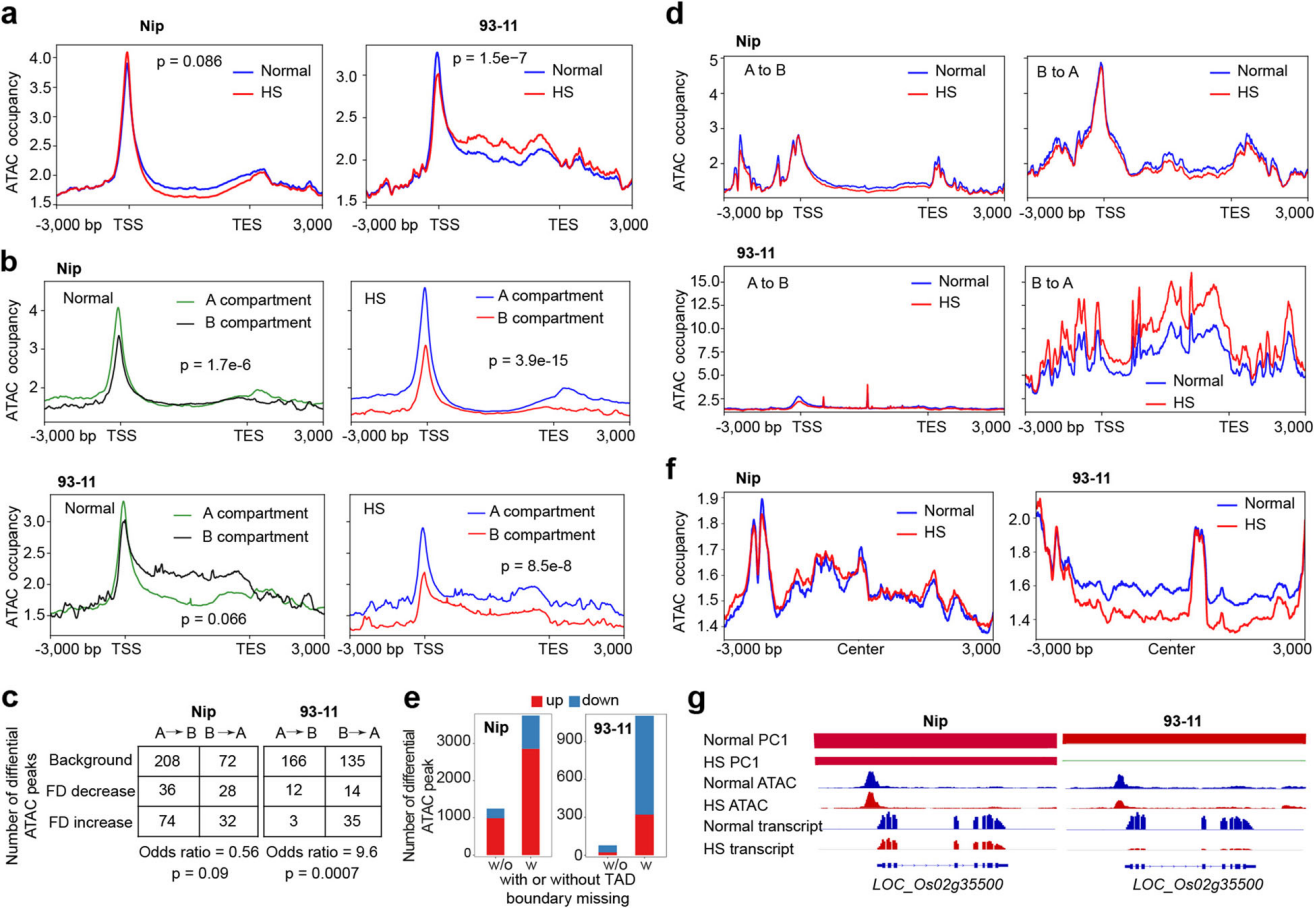
Chromatin 3D structure and chromatin accessibility are correlated upon HS in rice. **a** Distribution of ATAC peaks around genes and the changes upon HS in Nip and 93-11, respectively. ATAC occupancy was defined as the normalized reads count in ATAC-seq data. *p* values were calculated by performing the Wilcoxon test. **b** Distribution of ATAC peaks around genes in A/B compartment and the changes upon HS in Nip and 93-11, respectively. *p* values were calculated by performing the Wilcoxon test. **c** Relationship between A/B compartment and differential ATAC peaks. Tables showing the number of differential ATAC peaks in A/B compartment switch regions upon HS in Nip and 93-11, respectively. Odds ratio indicates the strength of the association, and *p* values that indicate the association significance were calculated by Fisher’s exact test. **d** Distribution of ATAC peaks around genes in A/B compartment switching regions and the changes upon HS in Nip and 93-11, respectively. **e** Comparing of the number of differential ATAC peaks between domain with and without (w/o) missed TAD boundary upon HS in Nip and 93-11, respectively. **f** Distribution of ATAC peaks around TADs and the changes upon HS in Nip and 93-11, respectively. **g** Example showing the correlation of Hi-C PC1, ATAC signal, and RNA-seq reads upon HS. In 93-11, *LOC_Os02g35500* located in the A compartment under normal condition and switched to the B compartment upon heat stress, associated with its reduced ATAC peak and downregulated gene expression. In Nip, *LOC_Os02g35500* located in the A compartment under normal and HS condition, and ATAC peak and gene expression were not significantly changed upon HS

To determine whether changes in higher-order organization are correlated with local changes in chromatin state, we performed Assay for Transposase-Accessible Chromatin sequencing (ATAC-seq), which captures accessible regions in the genome, thus measuring the active and inactive chromatin, under normal and HS conditions for Nip and 93-11, respectively. ATAC-seq data were generated from two replicates for each sample with high Pearson correlation coefficients (Additional file 1: Fig. S4a,b), suggesting reproducible detection of chromatin accessibility. Considering the ATAC peaks were not well characterized in rice, we compared the gene expression levels between the genes with and without ATAC peaks. As expected, in all the samples, genes with ATAC signals at promoter or gene body were significantly higher expressed than those without ATAC peaks (Additional file 1: Fig. S4c). Interestingly, we found that ATAC signal significantly overlapped with DNA N^6^-methyldeoxyadenosine (6mA) sites (Additional file 1: Fig. S4d), which was reported as a new epigenetic marker and associated with HS response in rice [19, 22–24], and may suggest that local changes in chromatin state may involve in HS response as well. Next, we divided the genome into gene bodies, promoters, and intergenic regions to examine the differential ATAC peak distribution around functional elements and found most of the differential ATAC peaks located in the promoter region, and the percentage in the promoter is obviously higher in 93-11 than that in Nip (Additional file 1: Fig. S4e). Closer examination revealed that ATAC signals were enriched at the transcription start site (TSS). Interestingly, the TSS enrichment of ATAC was decreased in response to HS in both Nip and 93-11 and more significantly decreased in 93-11 than that in Nip (Fig. 3a). We also observed that the overall level of ATAC in the gene body was decreased in Nip versus increased in 93-11 (Fig. 3a), suggesting differential chromatin accessibility dynamics in gene body between Nip and 93-11 upon HS.

The remodeling of chromatin 3D organization affects the chromatin epigenetic state [25]. By integrating the chromatin organization changes with the ATAC data, we found that TSS enrichment of ATAC peaks in the A compartment was significantly higher than that in the B compartment under the normal conditions, and this difference was larger upon HS: TSS enrichment increased in the A compartment and decreased in the B compartment, and these changes were conserved between Nip and 93-11 (Fig. 3b), suggesting that the chromatin compartmentation more accurately reflects chromatin state around TSS under HS. Moreover, we found a distinct dynamic pattern in the gene body between Nip and 93-11. In Nip, similar ATAC levels were observed between A and B compartments in the normal condition, and only slightly increased ATAC levels were found in the A compartment versus the B compartment upon HS. In 93-11, we found that ATAC levels were higher in the B compartment than that in the A compartment in normal conditions, and ATAC levels became lower in the B compartment than that in the A compartment upon HS (Fig. 3b). Further analysis suggested that differential ATAC peaks significantly associate with A/B compartment transition (Fig. 3c). As expected, we found even more distinct features between Nip and 93-11 in these transition regions. First, the distribution patterns of ATAC peaks were clearly different. In Nip, we found several peaks including a major ATAC enrichment at TSS, and no obvious difference between normal and HS conditions. However, in 93-11, the ATAC enrichment was largely decreased in the A-B region but was overall increased in the B-A region upon HS (Fig. 3d). These changes agreed with the expression data that DEGs in 93-11 displayed significantly more downregulation in the A-B transition region and upregulation in the B-A transition region than that in Nip. In addition, we found that most of differential ATAC peaks present in the domains with missed TAD boundary upon HS (Fig. 3e). Next, we looked closer and found no obvious changes in ATAC levels in the regions that lost TAD boundaries in Nip versus largely decreased ATAC levels in the regions that were losing TAD boundaries in 93-11 (Fig. 3f), suggesting the enlargement of TAD is associated with loss of chromatin accessibility. Interestingly, we found that the chromatin changes affect the expression of stress-related genes. For example, *LOC_Os02g35500* encodes glyoxylate reductase, which is associated with many stresses, including HS [26]. In 93-11, downregulation of *LOC_Os02g35500* was associated with the A-B transition and decreased of ATAC signals upon HS. By contrast, in Nip, in the corresponding loci, the chromatin was largely unchanged and the expression change of *LOC_Os02g35500* was much smaller upon HS (Fig. 3g). Although we could not exclude the possibility that chromatin interactions could be modified due to local gene expression changes, the example also implies that a correlation among 3D chromatin structure, chromatin accessibility, and gene expression level might contribute to regulating the response to HS in rice.

## Conclusions

In summary, this study provides a comprehensive integration of Hi-C, RNA-seq, and ATAC-seq of two rice cultivars Nip and 93-11, which demonstrates the reorganization of local and global chromatin structure in response to heat stress. The chromatin accessibility and 3D organization were correlated and consistent with the expression dynamics. The distinct chromatin reorganization and the correlated gene expression between Nip and 93-11 emphasize the importance of the 3D genome as a new layer to understanding stress response in plants.

## Methods

### Plant materials

Rice plants were grown on Yoshida solution in a growth chamber under short-day (SD) conditions (10 h light/14 h dark) with a light intensity of 800 μmol m^−2^ s^−1^. To test heat response, we incubated 3-week-old Nip and 93-11 seedlings grown in culture solution 45 °C for 36 h in a growth chamber with high relative humidity (> 80%) and low light intensity (60–80 μmol m^−2^ s^−1^). Then the aerial parts of these seedlings were harvested for Hi-C, RNA-seq, and ATAC-seq library preparation.

### Hi-C experiments and sequencing

Hi-C experiments were performed as described previously with some modifications [9]. Briefly, 2 g of the aerial parts of the rice seedlings was fixed with formaldehyde and lysed and grounded into powder in liquid nitrogen. The procedure for the Hi-C experiment, including chromatin digestion, labeling of DNA ends, DNA ligation, purification, and fragmentation, was performed as described previously [27]. The cross-linked DNA was digested with Dpn II restriction enzyme by incubating overnight at 37 °C. DNA ends were labeled with biotin and incubated at 37 °C for 45 min, and the enzyme was inactivated with 20% SDS solution. DNA ligation was performed by the addition of T4 DNA ligase (Fermentas) and incubation at 4 °C for 1 h followed by incubation at 22 °C for 4 h. After ligation, proteinase K was added to reverse cross-linking during incubation at 65 °C overnight. DNA fragments were purified and dissolved in 86 μl of water. Unligated ends were then removed. Purified DNA was fragmented to a size of 300–700 bp using a sonicator (Covaris S220), and DNA ends were then repaired. DNA fragments labeled by biotin were finally separated on Streptavidin C1 beads (Life Technologies). Chimeric fragments representing the original cross-linked long-distance physical interactions were then processed into paired-end sequencing libraries. Hi-C libraries were constructed using the Illumina TruSeq DNA Sample Prep Kit and were sequenced on an Illumina HiSeq X-ten platform (Illumina, USA) with 2 × 150-bp reads. Hi-C experiments and sequencing were performed with two biological replicates.

### Hi-C data analysis

Hi-C data were preprocessed using the streamlined pipeline HiC-pro [28]. Briefly, sequencing reads were mapped to the reference genome by Bowtie2 in –very-sensitive-local mode. Mapped reads were paired and pairs with multiple hits, low MAPQ, self-circle, and PCR duplicates were removed. Output files containing all valid pairs were used for subsequent analysis.

HiCExplorer was used to create the contact matrices [20], and the genomes were divided into bins of unequal size demarcated by the genomic positions of the restriction site and a matrix was created with these bins as rows and columns. The mapped reads were processed to count the number of times any two bins were connected by the Hi-C reads pairs. The following reads were discarded: read pairs that were not uniquely mapped, were duplicated, and contained a dangling end indicative of defective religation.

Chromosomal compartments were identified using principal component analysis (PCA) on contact maps at 100-kb resolution. We performed PCA analysis by using runHiCpca.pl script in HOMER Package. Briefly, we first identified the best background model for distance normalized contact normalization. Distance matrix was generated for each chromosome, and correlations between the contact profiles from each region against each other region were calculated. Then the value of the first principal components from the correlation matrix was obtained. With the rice genome annotation file, the active regions were assigned. The first component typically represents the compartment profile in genome—positive eigenvector value enriches with the A compartment (gene-rich regions) and negative eigenvalue enriched with the B compartment (gene-poor regions).

TAD calling worked in two steps: First, we used HiCExplorer to compute a TAD-separation score based on a *z*-score matrix for all bins. Next, bins with a local minimum of the TAD-separation score were evaluated with respect to the surrounding bins to assign a *p* value. A cutoff was applied to select the bins more likely to be TAD boundaries.

To call TADs from Hi-C data, we used an approach based on the TAD-separation score, using hicFindTADs from HiCExplorer as described previously [18] with following parameter: --minDepth 15,000 --maxDepth 150000 --step 5000 --thresholdComparisons 0.05 --delta 0.01 --correctForMultipleTesting fdr at 5-kb resolution. We calculated TAD-separation score profiles with different window sizes, ranging from 15 to 30 kb by 5-kb step. The TAD-separation score was calculated as the average *z*-score of all Hi-C contact bins between an adjacent window upstream and an adjacent window downstream. Bins with a local minimum of the TAD-separation score were evaluated with respect to the surrounding bins to assign a *p* value. Next, delta < 0.01 and thresholdComparisons < 0.05 were applied to select the bins more likely to be TAD boundaries.

For data visualization, only reads mapping to unique genomic positions were selected. The visualization was performed using deepTools [29].

### RNA sequencing and expression quantification

RNA-seq experiments were performed as described previously with some modifications [30]. Briefly, three biological replicates were designed for each RNA-seq experiment of Nip and 93-11. RNAs from aerial parts of normal and HS treatment seedlings were extracted using the RNeasy Plus Mini Kit (Qiagen). The extracted RNA was purified using poly-T oligo-attached magnetic beads. All transcriptome libraries were constructed using the Illumina TruSeq library Stranded mRNA Prep Kit and then sequenced on the Illumina HiSeq X-ten platform. RNA-seq raw reads were quality-checked by FastQC and aligned onto the rice (Nip/93-11) genomes using STAR [31] with the following parameters: --outFilterMultimapNmax 20 --outSAMtype BAM SortedByCoordinate --outSAMstrandField intronMotif. Then featureCounts (v1.6.3) was used to quantify reads generated from bam files [32]. DESeq2 was employed and used to evaluate differential gene expression between normal and HS conditions [33]. Genes with an absolute value of log2 ratio ≥ 1 and adjust *p* value < 0.01 were defined as DEGs. Gene expression levels were estimated using FPKM values. Gene Ontology and KEGG pathway enrichment analysis were performed with R package clusterProfiler [34].

### Nucleus isolation and ATAC-seq

Two biological replicates were collected for both normal and HS samples, respectively. Purification of nuclei using the method was performed as described previously with the following modifications [35, 36]. Approximately 0.5 g freshly harvested aerial parts of the normal and HS treatment seedlings were collected for nucleus isolation. The samples were collected and immediately chopped in 2 ml of pre-chilled lysis buffer (15 mM Tris-HCl pH 7.5, 20 mM NaCl, 80 mM KCl, 0.5 mM spermine, 5 mM 2-ME, 0.2% Triton X-100), respectively. Next, the total mixture was filtered using Miracloth twice and loaded onto the surface of 2-ml dense sucrose buffer in a 15-ml Falcon tube. The nuclei were pelleted by centrifugation at 1000*g* at 4 °C for 10 min, and the pellets were resuspended in 500 ml pre-chilled lysis buffer. After checking the quality of nuclei under a microscope using a DAPI channel, the nuclei were washed with Tris-Mg buffer (10 mM Tris-HCl pH 8.0, 5 mM MgCl_2_) once and the supernants removed. Freshly purified nuclei to be used for ATAC-seq were kept on ice prior to the transposase integration reaction. Transposase integration reactions and sequencing library preparations were then performed as previously described [35]. In brief, 50 ng of genomic DNA was used in each 50 μl Tn5 transposase integration reaction for 30 min at 37 °C using Nextera reagents. DNA fragments were purified using the Minelute PCR purification kit (Qiagen), eluted in 11 μl elution buffer, and the entirety of each sample was then amplified using High Fidelity PCR Mix (NEB) and barcode primers for 9-12 PCR cycles. These amplified ATAC-seq libraries were purified using AMPure XP beads (Beckman Coulter), quantified by qPCR with the NEBNext Library Quantification Kit (NEB), and analyzed on a Bioanalyzer High Sensitivity DNA Chip (Agilent) prior to pooling and sequencing. A Qubit fluorometer and Agilent Bioanalyzer were used to check library quality and concentration. DNA libraries were constructed using NEBNext DNA Library Prep Kit (Neb) and sequenced on an Illumina HiSeq X-ten platform with 150-bp paired-end reads.

### ATAC-seq data analysis

Sequencing reads were first trimmed with Nextera adapter sequences by Cutadapt (version 2.4 with Python 3.7.3) and then were mapped to the reference genome using Bowtie2 software (version 2.3.5.1) with default parameters [37, 38]. Reads with mapping quality (phred scale) < 30 or not properly paired or mapped to the chloroplast or mitochondrial genomes were filtered using SAMtools (version 1.9). Duplicated reads from PCR amplification were further removed with Picard MarkDuplicates (version 2.20.3). Narrow peaks were called using MACS2 (version 2.1) with an adjusted *p* value cutoff as 0.05 [39]. Differential peaks between HS and control samples were called with R package DiffBind (version 2.12.0 with R 3.6.0) [40]. Differential ATAC peak was defined at a cutoff adjusted *p* value < 0.05.

### Statistical analysis

The significance of the difference between two groups was determined by the Wilcoxon test. Other statistical analyses were indicated in the relevant figure legends.

## Supplementary Information

**Additional file 1: Figure S1.** Hi-C of rice Nip and 93-11 in normal and heat stress condition. **Figure S2.** Chromatin 3D structure dynamics in response to heat stress in rice Nip and 93-11. **Figure S3.** Gene expression analysis rice Nip and 93-11 upon HS. **Figure S4.** ATAC-seq of rice Nip and 93-11 in normal and HS conditions.

**Additional file 2: Table S1.** Statistics Hi-C sequencing. **Table S3.** Statistics RNA-seq. **Table S4.** List of differentially expressed genes upon HS treatments in Nip. **Table S5.** List of differentially expressed genes upon HS treatments in 93-11.

**Additional file 3: Table S2** List of TADs identified in normal and HS conditions in Nip and 93-11, respectively.

## Data Availability

The Hi-C, ATAC-seq, and RNA-seq data in this study have been deposited in the NCBI Gene Expression Omnibus (GEO) database with the accession numbers: GSE144564 [41], GSE144565 [42], and GSE144566 [43]. The related scripts, including some scripts modified from [44], were uploaded into GitHub at https://github.com/guxiaofengcaas/publiccode.

## Abbreviations

ATAC-seq: Assay for Transposase-Accessible Chromatin sequencing
DEG: Differentially expressed gene
GO: Gene Ontology
HS: Heat stress
PCA: Principal component analysis
RNA-seq: RNA sequencing
FPKM: Fragments per kilobase per million mapped reads
TAD: Topologically associating domain
TSS: Transcription start site
TTS: Transcription termination site
3D: Three-dimensional
6mA: N^6^-methyldeoxyadenosine
