# Supplementary material for: Reorganization of the 3D chromatin architecture of rice genomes during heat stress
Source: BMC Biol. 2021 Mar 19;19:53. doi: 10.1186/s12915-021-00996-4 (PMC7977607; doi:10.1186/s12915-021-00996-4)
Supplement: Supplementary file 1 — Additional file 1: Figure S1. Hi-C of rice Nip and 93-11 in normal and heat stress condition. Figure S2. Chromatin 3D structure dynamics in response to heat stress in rice Nip and 93-11. Figure S3. Gene expression analysis rice Nip and 93-11 upon HS. Figure S4. ATAC-seq of rice Nip and 93-11 in normal and HS conditions. [file 12915_2021_996_MOESM1_ESM.docx]

**Additional file 1**

**Reorganization of the 3D chromatin architecture of rice genomes during heat stress**

Zhe Liang^1^^,2†^, Qian Zhang^1†^, Changmian Ji^3^, Guihua Hu^1^, Pingxian Zhang^1^, Yifan Wang^1^, Liwen Yang^1^ and Xiaofeng Gu^1^*

*Correspondence: guxiaofeng@caas.cn

^†^Zhe Liang and Qian Zhang contributed equally to this work.

^1^Biotechnology Research Institute, Chinese Academy of Agricultural Sciences, Beijing, 100081, China.

^2^Centre for Organismal Studies, Heidelberg University, Heidelberg, 69120, Germany

^3^Institute of Tropical Bioscience and Biotechnology, Chinese Academy of Tropical Agricultural Sciences, Haikou, 571101, China


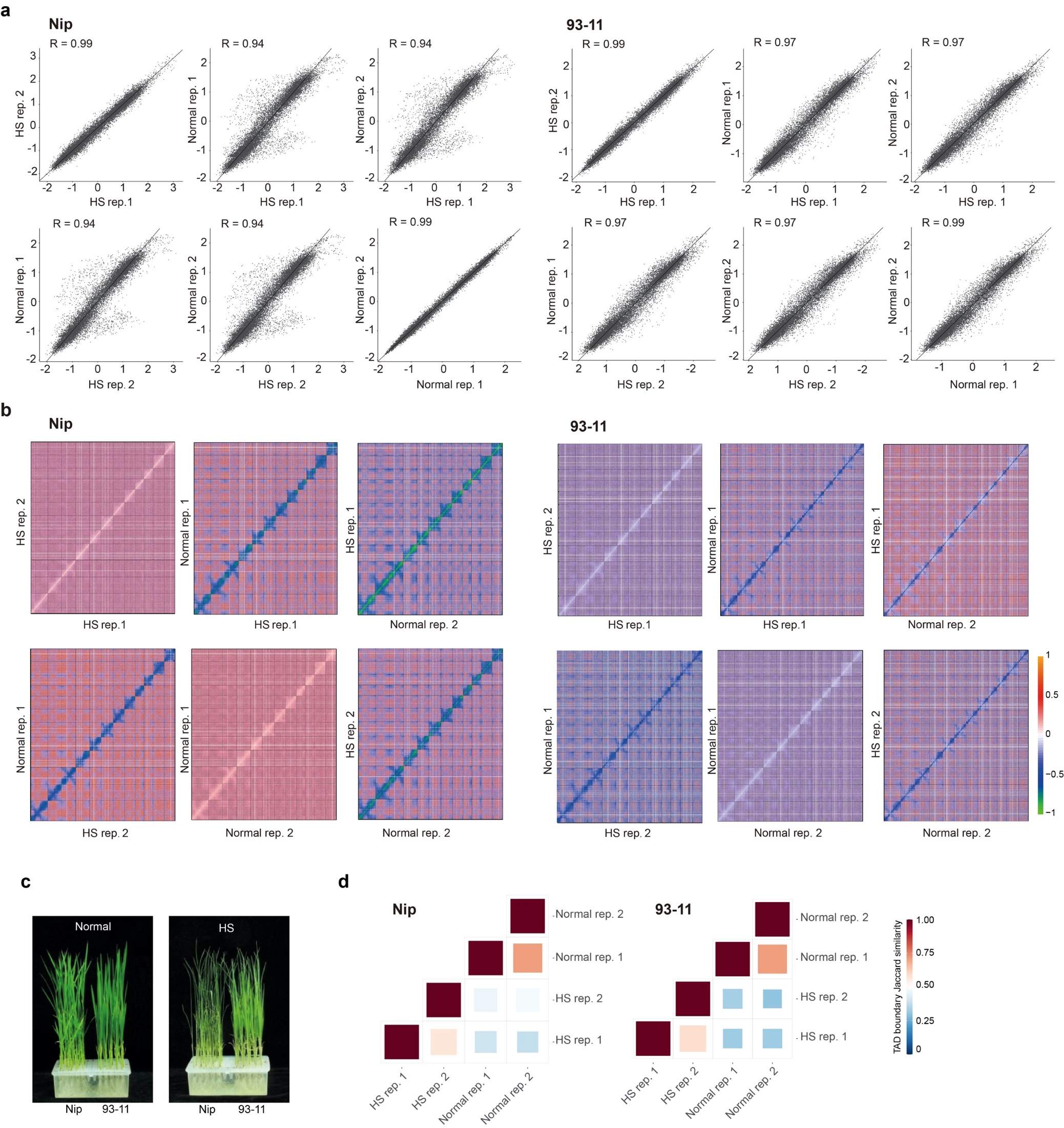


**Additional file 1: Figure S1.** Hi-C of rice Nip and 93-11 in normal and heat stress condition. **a** Scatter plots showing pairwise comparison of comparing log10 number of normalized interaction values for normal and HS samples for Nip and 93-11, respectively. R, Pearson correlation coefficient. **b** Heatmaps showing pairwise comparison of relative differences of interaction frequencies between normal and HS samples for Nip and 93-11, respectively. Colour bar, relative difference of normalized interaction number. **c** Phenotypes of Nip and 93-11 seedlings under HS. The 93-11 seedlings showed a greater tolerance to HS than did Nip seedlings. **d** Heatmap showing TAD boundary similarity. The Jaccard similarity coefficient was counted by using the number of shared boundaries (intersection) divide the total boundaries over both samples (union).


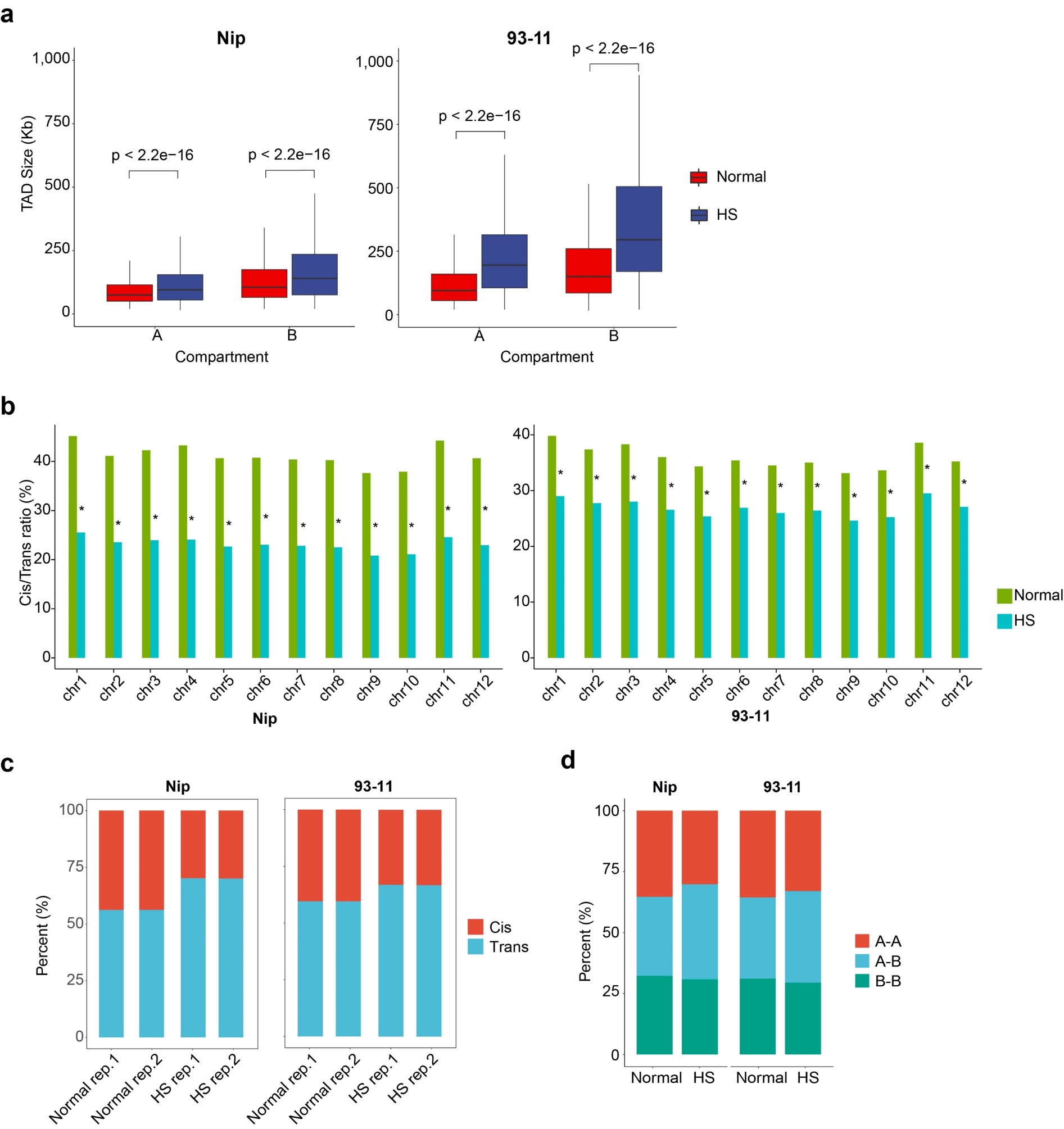


**Additional file 1: Figure S2.** Chromatin 3D structure dynamics in response to heat stress in rice Nip and 93-11. **a** Boxplot comparing TAD size in A/B compartment between normal and HS samples in Nip and 93-11, respectively. p values were calculated by performing two-tailed unpaired Student's *t*-test. **bc** Comparison of cis interactions to trans interactions between normal and HS samples in Nip and 93-11, respectively, for each chromosome (**b**) and each replicate (**c**). Asterisks indicate significant differences (two-tailed unpaired Student's *t*-test, p < 10−5). **d** Changes of percentage of three types of interaction (A-A, B-B, A-B) upon HS in Nip and 93-11, respectively.


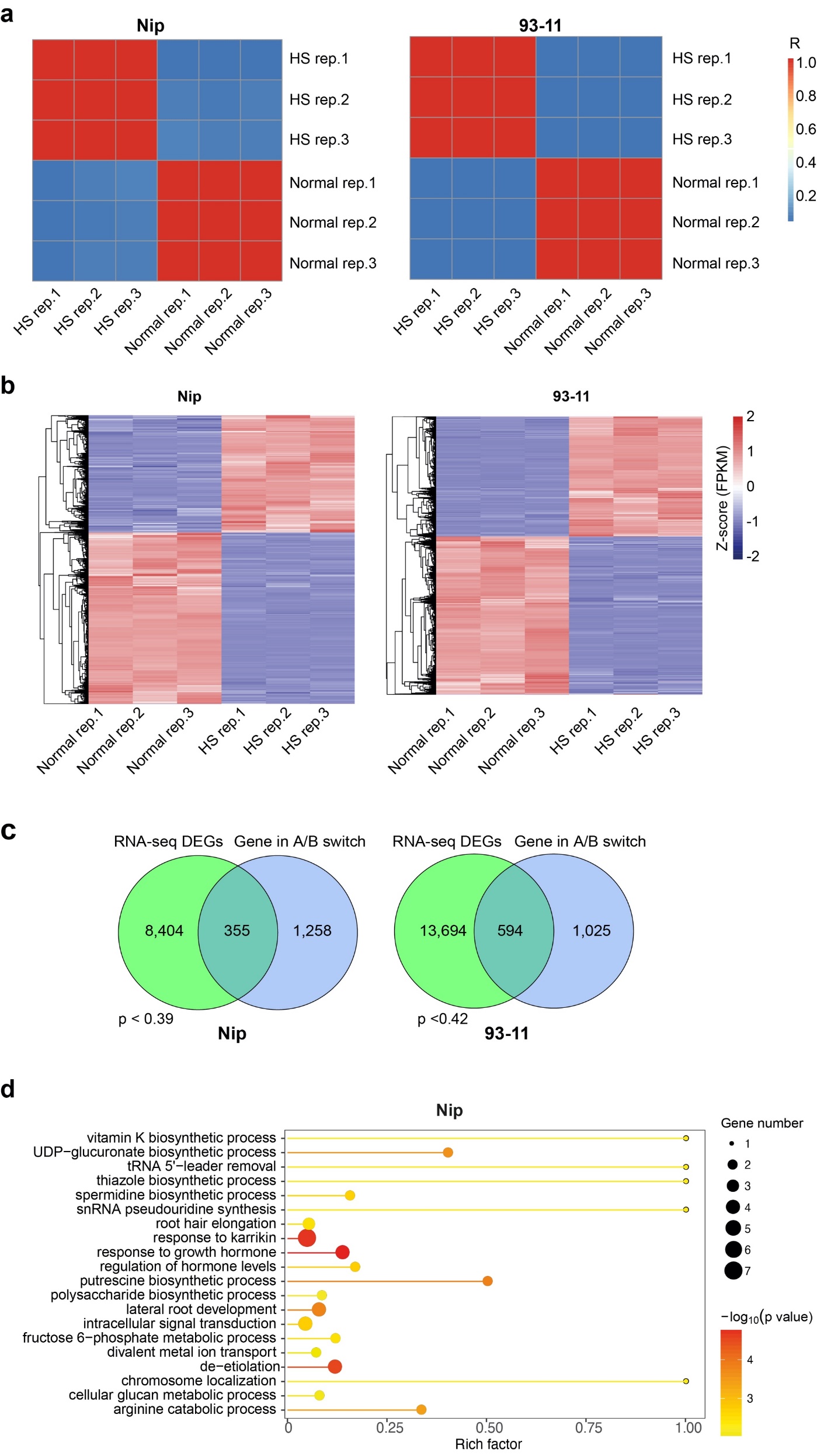


**Additional file 1: Figure S3.** Gene expression analysis rice Nip and 93-11 upon HS. **a** Heatmap showing Pearson correlations between RNA-seq experiments of normal and HS samples for Nip and 93-11, respectively. There were three biological replicates for each experiment. **b** Heat map showing differentially expressed genes (DEGs) (adjusted p < 0.01, fold change > 2) upon heat stress in Nip and 93-11, respectively. **c** Venn diagram comparing DEGs and genes located in altered A/B compartment. p values of the overlap between the two datasets were calculated by performing hypergeometric test with the total number of rice genes as the reference. **d** Scatter plots of significant biological processes as determined by GO enrichment analysis of DEGs in A-B or B-A transition region upon heat stress in Nip.


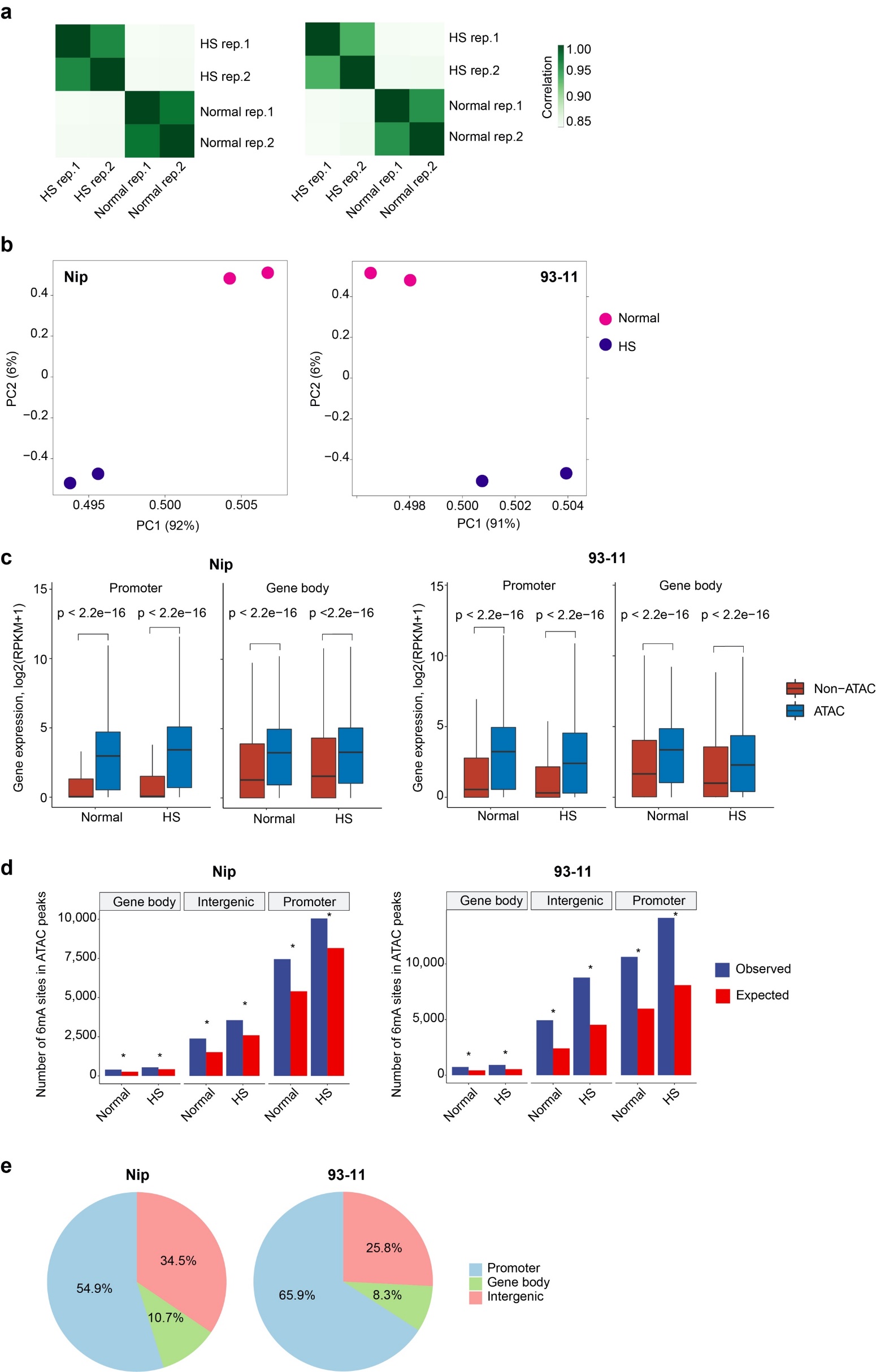


**Additional file 1: Figure S4.** ATAC-seq of rice Nip and 93-11 in normal and HS conditions. **a** Heatmap showing Pearson correlations between ATAC-seq experiments of normal and HS samples for Nip and 93-11. There were two biological replicates for each experiment. **b** PCA plot on the replicates of the ATAC-seq samples in Nip and 93-11, respectively. **c** Boxplot comparing gene expression between non-ATAC and ATAC marked promoter (2kb upstream of TSS) or gene body in Nip and 93-11, respectively. p values were calculated by performing two-sided Wilcoxon test. **d** Number of 6mA sites that overlapped with ATAC peaks within intergenic regions, promoters, and gene bodies. Asterisks indicate significant differences between observed 6mA sites and expected sites (binomial test, p < 10^−5^). **e** Distribution pattern of differential ATAC peaks within intergenic regions, promoters, and gene bodies.
